# Continuous, hyperfractionated, accelerated radiotherapy (CHART).

**DOI:** 10.1038/bjc.1989.64

**Published:** 1989-03

**Authors:** S. Dische, M. I. Saunders

**Affiliations:** Marie Curie Research Wing, Regional Radiotherapy and Oncology Centre, Mount Vernon Hospital, Northwood, Middlesex.


*Br. J. Cancer* (1989), **59**, 325–326
© The Macmillan Press Ltd., 1989

**EDITORIAL**

### Continuous, hyperfractionated, accelerated radiotherapy (CHART)

S. Dische & M.I. Saunders

*Marie Curie Research Wing, Regional Radiotherapy and Oncology Centre, Mount Vernon Hospital, Northwood, Middlesex HA6 2RN, UK.*

The main variables in a course of radiotherapy are the number of fractions, the dose per fraction, the total dose given and the overall duration of treatment. Radiotherapists endeavour to employ a combination which will achieve the maximum tumour control with the minimum of normal tissue damage. At each centre in the United Kingdom regimes are employed in curative treatment that are based upon clinical experience, practical considerations and local tradition. Although, in nearly all, daily treatment is given on five days of the week, the number of fractions ranges from 15 to 35, the individual dose from 1.8 to 3.4 Gy, the total dose from 50 to 70 Gy and the overall duration from 3 to 7 weeks.

An accumulation of data from the laboratory and from the clinic now allows some explanation of the relationship between these variables (Withers *et al.*, 1982, 1988; Trott & Kummermehr, 1984; Denekamp, 1986; Fowler, 1986; Tubiana, 1988). In human tumours it has been possible to determine, using flow cytometry, not only the percentage of cells in S phase, but also the duration of S phase, and hence the potential doubling time, by giving an intravenous administration of bromodeoxyuridine 4–8 h before tumour sampling. Using this technique half of the human tumours studied showed a potential to double their cell number in 5 or fewer days (Begg *et al.*, 1985, 1988; Wilson *et al.*, 1985, 1988). It is probable that the cells which survive the initial treatments of a course of radiotherapy will repopulate rapidly and replace those that have been killed. It follows that the longer the overall duration of treatment the greater must be the chance for repopulation to be a cause of failure. Turning to the normal tissues, we now know that the giving of a course of radiotherapy in many small individual doses may spare late damage, for injury to the critical supporting connective tissue is minimised under these conditions (Withers *et al.*, 1982; Thames, 1988). This knowledge may explain why different regimes may give similar tumour control and morbidity; however, it has also led to clinical exploration to develop new regimes which might bring a real advantage in tumour control.

In order to shorten the overall duration of radiotherapy and use a small individual dose per fraction, more than one treatment must be given on each day. There are now a number of completed and ongoing clinical studies using multiple treatments in one day and experience has already shown that a normal dose increment of 2 Gy cannot be maintained while giving accelerated treatment. There are problems with the tolerance of normal tissues and if a full tumour dose is given early reactions may not heal and will continue on to late morbidity (Peracchia & Salti, 1981; Van den Bogaert *et al.*, 1982; Dische & Saunders, 1988).

To overcome this, many workers have interrupted their accelerated treatment in order to allow for normal tissue recovery (Van den Bogaert *et al.*, 1982; Wang *et al.*, 1986; Dische & Saunders, 1988). The rest periods have commonly been between 2 and 4 weeks but these, and also the weekend gap between the last treatment on Friday and the first on Monday, may allow considerable repopulation by surviving tumour cells to occur.

A scheme of continuous, hyperfractionated, accelerated radiotherapy (CHART) was devised at Mount Vernon and took note of the biological data available (Saunders & Dische, 1986). Radiotherapy was given three times daily for a continuous period of 12 days. As treatment was commenced on a Monday this required continuation through one weekend. The time interval between treatments given on each day was of considerable importance. If it were too short the maximum repair of sub-lethal injury in normal tissues would not have occurred and as a result some of the benefit would have been lost; based on laboratory data a period of 6 hours was chosen (Saunders *et al.*, 1988). A pilot study which included 128 patients was commenced in January 1985 and completed in December 1987. An individual dose of 1.4 Gy was given to the first 38 patients so that a total minimum tumour dose of 50.4 Gy was achieved in the 36 treatments. As tolerance appeared good the individual dose was increased to 1.5 Gy so that the total dose given to the subsequent 90 patients was 54.0 Gy (Saunders & Dische, 1989, and unpublished).

Early radiation reactions have been well tolerated, but where the tongue has been included and the higher doses given, delays up to 6 months have occurred in a few cases before final healing. An interim assessment of late changes suggests that these may be less than after conventional treatment.

Included were 48 patients with advanced squamous cell cancer in the head and neck region who completed treatment. A complete regression was apparent in 44 (92%) of the 48 primary tumours and also in 18 (78%) of 23 patients who presented, in addition, secondary nodes. In an analysis performed in October 1988 concerning the 37 patients with $T_3$ and $T_4$ tumours when comparison was made with comparable previously treated cases at Mount Vernon there were statistically significant improvements in tumour control and survival (Saunders & Dische, unpublished).

The results using CHART in 52 patients with locally advanced carcinoma of the bronchus were also compared with a previously treated group of 62 patients with similar disease. Using CHART complete radiological disappearance of tumour was obtained in 22 (42%) of the 52 patients compared with 9 (15%) of the 62 patients in the previous group. A review of the data in September 1988 showed that 30 (67%) of 45 patients treated more than 1 year previously remained alive at 1 year; of 26 treated more than 2 years previously 11 (42%) were alive at the 2-year interval (Saunders & Dische, 1989). These findings can be compared with 44 and 12% obtained in the previous study, where results were comparable with those commonly recorded when locally advanced bronchogenic carcinoma is treated by radiotherapy (Perez *et al.*, 1982; Schaake-Konig *et al.*, 1983).

As a result of a joint Medical Research Council and Cancer Research Campaign initiative, supported by the D of H, the promise of the pilot study is now to be tested in multi-centre randomised trials in bronchial and in head and neck cancer. It is hoped that randomisation into the studies

Received 23 November 1988.

will begin early in 1989. If, in the phase III studies, CHART is shown to be superior to conventional radiotherapy then the result will have significance for the whole of oncology, as the capacity of human tumours to repopulate rapidly will have been confirmed. Advances should follow the reduction of the overall duration of management from initial surgery through to completion of radiotherapy and cytotoxic chemotherapy.

### References

BEGG, A.C., McNALLY, N.J., SHRIEVE, D.C. & KÄRCHER, H. (1985). A method to measure duration of DNA synthesis and the potential doubling time from a single sample. *Cytometry*, **6**, 620.

BEGG, A.C., MOONEN, L., HOFLAND, I., DESSING, M. & BARTELINK, H. (1988). Human tumour cell kinetics using a monoclonal antibody against iododeoxyuridine; intra-tumour sampling variations. *Radiother. Oncol.*, **11**, 337.

DENEKAMP, J. (1986). Cell kinetics and radiation biology. *Int. J. Radiat. Biol.*, **49**, 357.

DISCHE, S. & SAUNDERS, M.I. (1988). Current concepts in fractionation. Fractionation – a review of the clinical data. *Br. J. Radiol.*, Suppl. **22**, 84.

FOWLER, J.F. (1986). Potential for increasing the differential response between tumors and normal tissues: can proliferation rate be used? *Int. J. Radiat. Oncol. Biol. Phys.*, **12**, 641.

PERACCHIA, G. & SALTI, C. (1981). Radiotherapy with thrice-a-day fractionation in short overall time: clinical experiences. *Int. J. Radiat. Oncol. Biol. Phys.*, **7**, 99.

PEREZ, C.A., STANLEY, K. & GRUNDY, G. (1982). Impact of irradiation technique and tumor extent in tumor control and survival of patients with unresectable non-oat cell carcinoma of the lung. Report by the Radiation Therapy Oncology Group. *Cancer*, **50**, 1091.

SAUNDERS, M.I. & DISCHE, S. (1986). Radiotherapy employing three fractions in each day over a continuous period of 12 days. *Br. J. Radiol.*, **59**, 523.

SAUNDERS, M.I., DISCHE, S., FOWLER, J.F. & 7 others (1988). Radiotherapy employing three fractions on each of twelve consecutive days. *Acta Oncol. Fasc. 2*, **27**, 163.

SAUNDERS, M.I. & DISCHE, S. (1989). Continuous, hyperfractionated, accelerated radiotherapy in non-small cell carcinoma of the bronchus. *Br. J. Radiol.* (in the press).

SCHAAKE-KONIG, C., SCHUSTER-UITTERHOEVE, L., HART, G. & GONZALES GONZALES, D. (1983). Prognostic factors of inoperable localized lung carcinoma treated by high dose radiotherapy. *Int. J. Radiat. Oncol. Biol. Phys.*, **9**, 1023.

THAMES, H.D. JR. (1988). Early fractionation in radiotherapy. *Acta Oncol. Fasc. 2*, **27**, 89.

TROTT, K.-R. & KUMMERMEHR, K. (1984). What is known about tumour proliferation rates to choose between accelerated fractionation or hyperfractionation? *Radiother. Oncol.*, **3**, 1.

TUBIANA, M. (1988). Repopulation in human tumors. A biological background for fractionation in radiotherapy. *Acta Oncol. Fasc. 2*, **27**, 83.

VAN DEN BOGAERT, W., VAN DER SCHUEREN, E., HORIOT, J.-C. & 4 others (1982). The feasibility of high dose multiple fractionation and its combination with anoxic cell sensitizers in the treatment of head and neck cancer. *Int. J. Radiat. Oncol. Biol. Phys.*, **8**, 1649.

WANG, C.C., SUIT, H.S. & BLITZER, P.H. (1986). Twice-a-day radiation therapy for supraglottic carcinoma. *Int. J. Radiat. Oncol. Biol. Phys.*, **12**, 3.

WILSON, G.D., McNALLY, N.J., DUNPHY, E.P., KÄRCHER, H. & PFRAGNER, R. (1985). The labelling index of human and mouse tumours assessed by bromodeoxyuridine staining *in vitro* and *in vivo* and flow cytometry. *Cytometry*, **6**, 641.

WILSON, G.D., McNALLY, N.J., DISCHE, S. & BENNETT, M.H. (1988). Cell proliferation in human tumours measured by *in vivo* labelling with bromodeoxyuridine. *Br. J. Radiol.*, **61**, 419.

WITHERS, H.R., TAYLOR, J.M.G. & MACIEJEWSKI, B. (1988). The hazard of accelerated tumor clonogen repopulation during radiotherapy. *Acta Oncol. Fasc. 2*, **27**, 131.

WITHERS, H.R., THAMES, H.D. & PETERS, L.J. (1982). Differences in fractionation response of acutely and late-responding tissues. In *Progress in Radio-Oncology, Vol. II*, Kärcher, K.H., Kogelnick, H.D. & Reinartz, G. (eds) p. 287. Raven Press: New York.


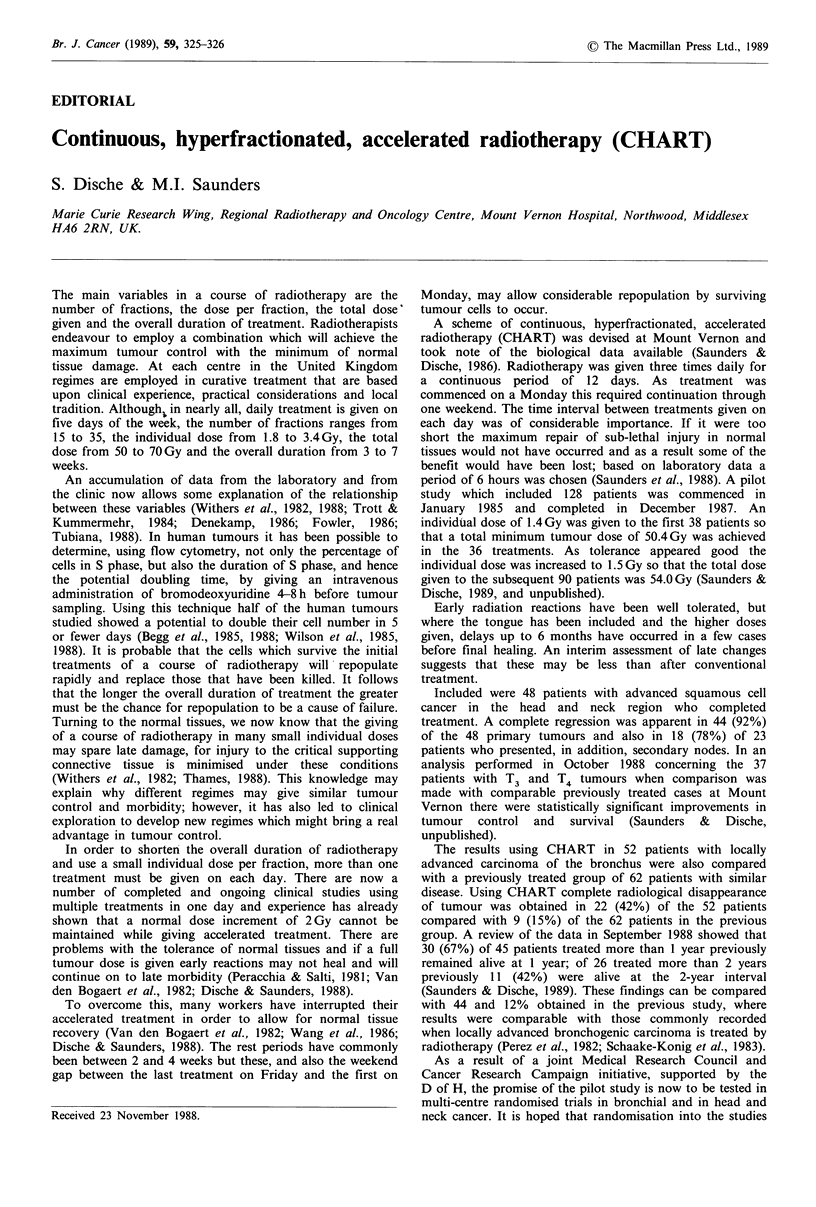

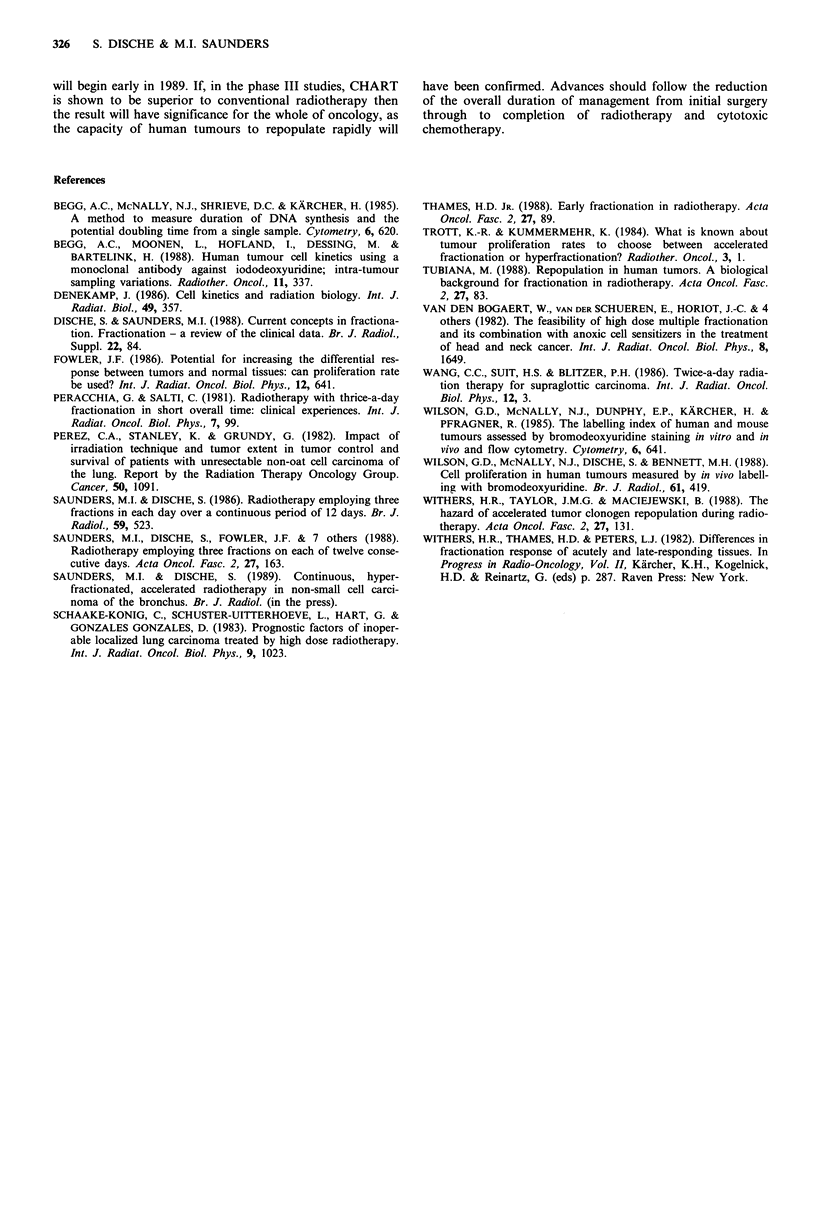


## References

[OCR_00158] Begg A. C., McNally N. J., Shrieve D. C., Kärcher H. (1985). A method to measure the duration of DNA synthesis and the potential doubling time from a single sample.. Cytometry.

[OCR_00162] Begg A. C., Moonen L., Hofland I., Dessing M., Bartelink H. (1988). Human tumour cell kinetics using a monoclonal antibody against iododeoxyuridine: intratumour sampling variations.. Radiother Oncol.

[OCR_00168] Denekamp J. (1986). Cell kinetics and radiation biology.. Int J Radiat Biol Relat Stud Phys Chem Med.

[OCR_00177] Fowler J. F. (1986). Potential for increasing the differential response between tumors and normal tissues: can proliferation rate be used?. Int J Radiat Oncol Biol Phys.

[OCR_00182] Peracchia G., Salti C. (1981). Radiotherapy with thrice-a-day fractionation in a short overall time: clinical experiences.. Int J Radiat Oncol Biol Phys.

[OCR_00187] Perez C. A., Stanley K., Grundy G., Hanson W., Rubin P., Kramer S., Brady L. W., Marks J. E., Perez-Tamayo R., Brown G. S. (1982). Impact of irradiation technique and tumor extent in tumor control and survival of patients with unresectable non-oat cell carcinoma of the lung: report by the Radiation Therapy Oncology Group.. Cancer.

[OCR_00199] Saunders M. I., Dische S., Fowler J. F., Denekamp J., Dunphy E. P., Grosch E., Fermont D., Ashford R., Maher J., Des Rochers C. (1988). Radiotherapy employing three fractions on each of twelve consecutive days.. Acta Oncol.

[OCR_00194] Saunders M. I., Dische S. (1986). Radiotherapy employing three fractions in each day over a continuous period of 12 days.. Br J Radiol.

[OCR_00209] Schaake-Koning C., Schuster-Uitterhoeve L., Hart G., Gonzalez Gonzalez D. (1983). Prognostic factors of inoperable localized lung cancer treated by high dose radiotherapy.. Int J Radiat Oncol Biol Phys.

[OCR_00219] Trott K. R., Kummermehr J. (1985). What is known about tumour proliferation rates to choose between accelerated fractionation or hyperfractionation?. Radiother Oncol.

[OCR_00229] Van den Bogaert W., van der Schueren E., Horiot J. C., Chaplain G., Arcangeli G., Gonzalez D., Svoboda V. (1982). The feasibility of high-dose multiple daily fractionation and its combination with anoxic cell sensitizers in the treatment of head and neck cancer. A pilot study of the Radiotherapy Group of the EORTC (European Organisation for Research on Treatment of Cancer).. Int J Radiat Oncol Biol Phys.

[OCR_00236] Wang C. C., Suit H. D., Blitzer P. H. (1986). Twice-a-day radiation therapy for supraglottic carcinoma.. Int J Radiat Oncol Biol Phys.

[OCR_00247] Wilson G. D., McNally N. J., Dische S., Bennett M. H. (1988). Cell proliferation in human tumours measured by in-vivo labelling with bromodeoxyuridine.. Br J Radiol.

[OCR_00241] Wilson G. D., McNally N. J., Dunphy E., Kärcher H., Pfragner R. (1985). The labelling index of human and mouse tumours assessed by bromodeoxyuridine staining in vitro and in vivo and flow cytometry.. Cytometry.

[OCR_00252] Withers H. R., Taylor J. M., Maciejewski B. (1988). The hazard of accelerated tumor clonogen repopulation during radiotherapy.. Acta Oncol.

[OCR_00068] van den Berg-Rust C. J. (1986). Uitslag Ewing-screening onvoldoende--en dan?. Ned Tijdschr Geneeskd.

